# Supplementary material for: Artificial lighting affects the landscape of fear in a widely distributed shorebird
Source: Commun Biol. 2023 Jan 31;6:131. doi: 10.1038/s42003-023-04486-x (PMC9889372; doi:10.1038/s42003-023-04486-x)
Supplement: Supplementary file 2 — Supplementary Information [file 42003_2023_4486_MOESM2_ESM.pdf]

Supporting information: Artificial lighting affects the landscape of fear in a widely distributed shorebird

Juho Jolkkonen[1], Kevin J. Gaston[2], Jolyon Troscianko[3*]

1. Department of Biological and Environmental Science, University of Jyväskylä, P.O. Box 35, 40014 University of Jyväskylä, Finland

2. Environment & Sustainability Institute, University of Exeter, Penryn Campus, Penryn, Cornwall TR10 9FE, UK.

3. Centre for Ecology & Conservation, University of Exeter, Penryn Campus, Penryn, Cornwall TR10 9FE, UK.
*Author for correspondence (e-mail: J.Troscianko@exeter.ac.uk)

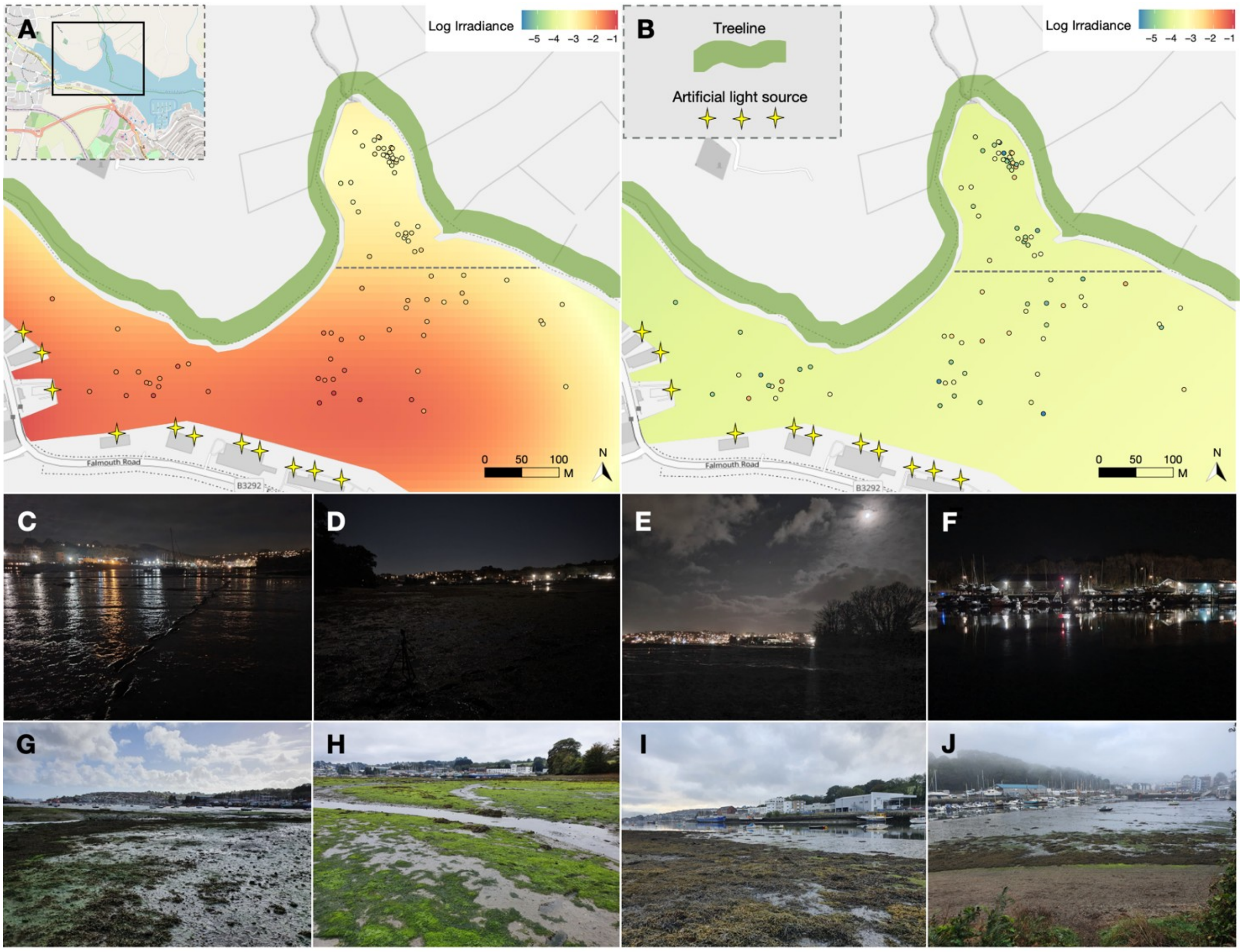

Fig S1. Nocturnal light environment varies within the study site

Top row: Heat maps showing variation in ALAN (A) and downwelling light (B) within the study site at night. Light environment measurements were taken from the flushing point of the approached curlew (points marked on the map, n = 86). The colour of the points represents the measured light intensity at the flushing point. Using these measurements, the heat map colour was modelled by explaining the light intensities with latitude and longitude. Within the study site, intensities of anthropogenic light primarily depended on the distance from the artificial light sources (e.g., streetlights and warehouses) that were directly illuminating the tidal flats on the south bank of the Penryn River (A). Further away from the light sources, towards the bottom of the narrow bay (‘creek’), separated by a dashed line (latitude 50.16873 °N), intensities of the horizontal ALAN were lower, creating a gradual variation of artificial illumination within the study site. Downwelling irradiance (B) from directly overhead, composed primarily of natural light, but also including some reflected ALAN sky glow on cloudy nights. As this measurement excluded the direct low-angle ALAN, location did not create any variation in downwelling light. Background map: Open Street Maps (OSM, http://www.openstreetmap.org/, accessed 7.12.2021. Middle row: Photographs (C-F) taken at different parts of the tidal flats showing the variation in horizontal ALAN and downwelling light in different parts of the tidal flats at night, facing south (Juho Jolkkonen, 2021). Bottom row: Daytime photographs (G-J) taken at different parts of the tidal flats facing south (Juho Jolkkonen, 2021).

Table S1. Variation in the horizontal ALAN and downwelling light intensity within the study site.

Irradiance ($cd{\cdot}m^{-2}$)	Min	Max	Mean	StDev
Horizontal ALAN	0.019	0.408	0.114	0.091
Downwelling light	0.003	0.130	0.030	0.030

Table S2. Results of the full factorial model explaining curlew's FID.

Horizontal ALAN, downwelling light, flock size and time until the low tide were significant predictors of the FID also in the full factorial model.

Variable	Estimate	SE	*t*	*p*
(Intercept)	56.63	1.31	43.29	**< 0.001**
Horizontal ALAN, linear	78.68	28.09	2.80	**0.007**
Horizontal ALAN, polynomial	-23.26	13.81	-1.68	0.096
Downwelling light, linear	46.94	17.08	2.75	**0.008**
Downwelling light, polynomial	22.22	13.54	1.64	0.105
Flock size	-4.30	1.97	-2.18	**0.033**
Temperature	-2.43	2.15	-1.13	0.261
Latitude	-2.29	3.18	-0.72	0.473
Longitude	-0.71	1.66	-0.43	0.671
Day	-2.16	2.36	-0.92	0.362
Time until low tide, linear	-11.60	13.32	-0.87	0.387
Time until low tide, polynomial	40.59	13.53	3.00	**0.004**
Low tide height, linear	26.64	14.46	1.84	0.070
Low tide height, polynomial	-17.53	17.02	-1.03	0.306

Table S3. Results of the best model explaining curlew's FID in the 'creek' including backlight as an explanatory variable.

Only downwelling light intensity explained variation in the FID in the 'creek' area where the levels of horizontal ALAN were low.

Variable	Estimate	SE	*t*	*p*
(Intercept)	37.23	5.37	6.94	**< 0.001**
Horizontal ALAN, linear	24.74	15.37	1.61	0.118
Horizontal ALAN, polynomial	3.42	11.95	0.29	0.777
Downwelling light, linear	50.01	14.96	3.34	**0.002**
Downwelling light, polynomial	19.67	11.38	1.73	0.095
Backlight, yes	0.60	4.81	0.12	0.902
Group size	-2.87	2.44	-1.17	0.250
Time until low tide, linear	-9.69	12.05	-0.80	0.428
Time until low tide, polynomial	23.91	13.26	1.80	0.082
Low tide height, linear	3.96	4.11	0.97	0.343

<u>Nocturnal vs diurnal FID</u>

Curlew foraging on the tidal flats took flight at a shorter distance at night than in the daytime when approached (Welch's t-test: $t = 7.70$, $df = 43.31$, $p < 0.001$; Fig S2A). This agrees with observations by Tillman (2009), who found that grey partridge flushed at closer distances at night. In the day-time, curlew FID varied between 57 and 143 m ($\bar{x} = 91.52 \pm 20.60$ m, n = 27), whereas at night, curlew escaped from 18 m up to 113 m distance ($\bar{x} = 56.63 \pm 20.45$ m, n = 86). Next, we analysed whether natural illumination from the moonlight explained any variation in nocturnal FID. We found that on moonless nights (i.e., during the New Moon phase or when the Moon was below the horizon), FID was shorter (18–83 m, $\bar{x} = 50.64 \pm 19.74$ m, n = 42) compared to nights when the moonlight illuminated the tidal flats (28–113 m, $\bar{x} = 62.34 \pm 19.67$ m, n = 44). The difference between moonlit and moonless nights was significant (Welch's t-test: $t = -2.75$, $df = 83.79$, $p = 0.007$; Fig S2A).

Effect of group size on FID

Group size is a major factor known to affect perceived predation risk and FID. Typically, a larger group size increases vigilance due to more eyes searching for the predators, which often increases FID[1-4]. Alternatively, the protective effect of the larger group sizes reduces the risk of being predated, predicting shorter FID[5-7]. At night, curlew foraging in larger flocks had shorter FIDs (Fig S2B; no Moon: $F_{2,39} = 17.02$, $p < 0.001$, Moon: $F_{1,42} = 14.08$, $p < 0.001$). This supports the latter theory of the larger group size's protective effect reducing the perceived predation risk[5-7]. However, flock size did not have a significant effect on FID in daylight ($F_{1,25} = 0.53$, $p = 0.474$; Fig S2B). Though we note that our daytime sample size was comparatively small to assess this effect. Curlew were also less frequent on the mudflats in the daytime, as the majority of the individuals were often foraging on the nearby fields. Thus, any flocks larger than 10 individuals were not tested in the daytime.

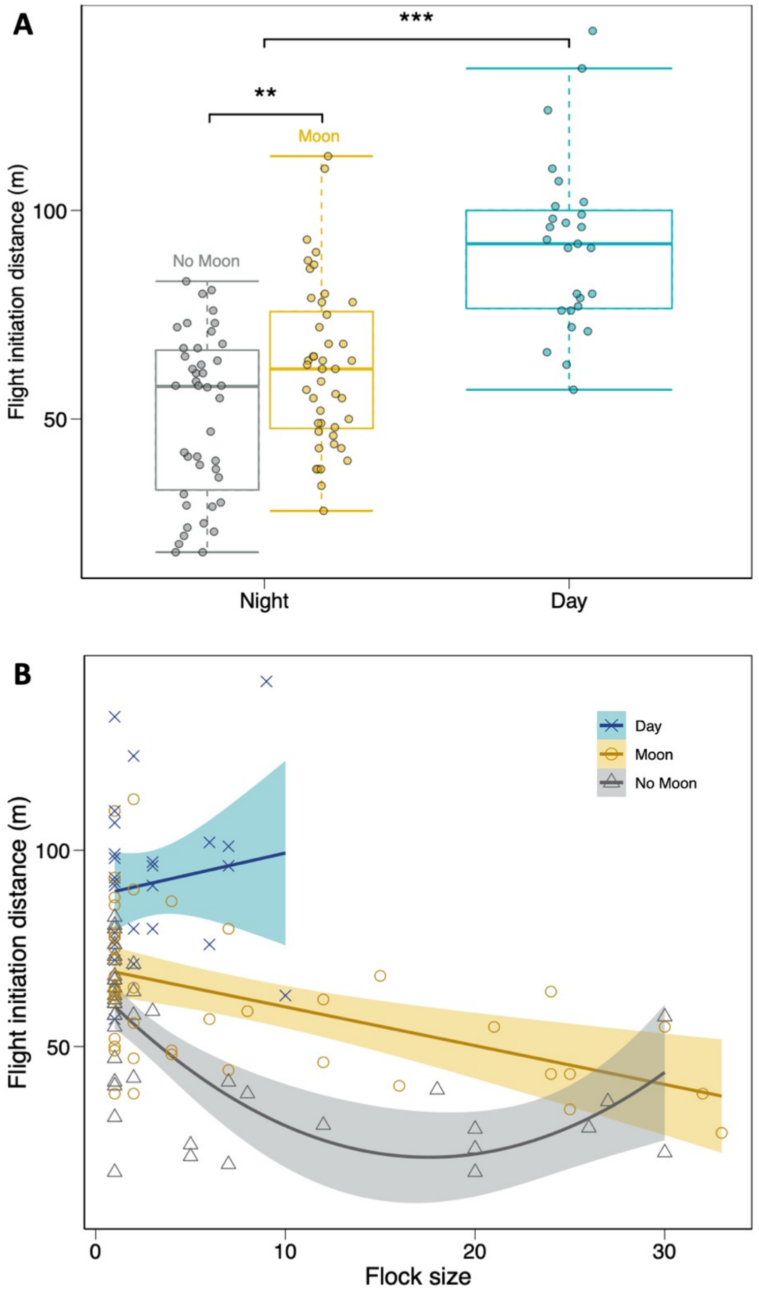

Fig S2. Natural light levels and flock size affect FID

(A) shows that curlew had significantly shorter FID at night than in the daytime. Similarly, FID was shorter at moonless nights compared to moonlight nights. Welch's t-test was used to calculate the difference between the groups (significance codes: '***' < 0.001 < '**' < 0.01). (B) shows the protective effect of larger groups shortening FID at night (both moonless and moonlight) but not in the daytime.

Supporting information references:

1. Blumstein, D. T. Developing an evolutionary ecology of fear: how life history and natural history traits affect disturbance tolerance in birds. *Anim. Behav.* **71**, 389–399 (2006).

2. Collop, C. *et al.* Variability in the area, energy and time costs of wintering waders responding to disturbance. *Ibis* **158**, 711–725 (2016).

3. Laursen, K., Kahlert, J. & Frikke, J. Factors affecting escape distances of staging waterbirds. *Wildl. Biol.* **11**, 13–19 (2005).

4. Morelli, F. *et al.* Contagious fear: Escape behavior increases with flock size in European gregarious birds. *Ecol. Evol.* **9**, 6096–6104 (2019).

5. Alexander, R. D. The Evolution of Social Behavior | Annual Review of Ecology, Evolution, and Systematics. *Annu. Rev. Ecol. Syst.* **5**, 325–383 (1974).

6. Pulliam, H. R. On the advantages of flocking. *J. Theor. Biol.* **38**, 419–422 (1973).

7. Ydenberg, R. C. & Dill, L. M. The Economics of Fleeing from Predators. *Adv. Study Behav.* **16**, 229–249 (1986).
