# Supplementary material for: Artificial lighting affects the landscape of fear in a widely distributed shorebird
Source: Commun Biol. 2023 Jan 31;6:131. doi: 10.1038/s42003-023-04486-x (PMC9889372; doi:10.1038/s42003-023-04486-x)
Supplement: Supplementary file 3 — Description of Additional Supplementary Files [file 42003_2023_4486_MOESM3_ESM.pdf]

Description of Additional Supplementary Files

File name: Supplementary Data
Description: Zip file contains the raw data and R script used to analyse the data and generate graphs. The R script also contains additional statistical output and model verification information.

File name: Video S1
Description: Thermal imaging video of a fox on the mudflats in the vicinity of the curlew

File name: Video S2
Description: Thermal imaging video of a fox on the mudflats in the vicinity of the curlew
