# Supplementary material for: Artificial lighting affects the landscape of fear in a widely distributed shorebird
Source: Commun Biol. 2023 Jan 31;6:131. doi: 10.1038/s42003-023-04486-x (PMC9889372; doi:10.1038/s42003-023-04486-x)
Supplement: Supplementary file 7 — Reporting Summary [file 42003_2023_4486_MOESM7_ESM.pdf]

nature portfolio

Corresponding author(s): Jolyon Troscianko

Last updated by author(s): 13/12/2022

Reporting Summary

Nature Portfolio wishes to improve the reproducibility of the work that we publish. This form provides structure for consistency and transparency in reporting. For further information on Nature Portfolio policies, see our Editorial Policies and the Editorial Policy Checklist.

Statistics

For all statistical analyses, confirm that the following items are present in the figure legend, table legend, main text, or Methods section.

n/a	Confirmed	
☐	☒	The exact sample size (*n*) for each experimental group/condition, given as a discrete number and unit of measurement
☐	☒	A statement on whether measurements were taken from distinct samples or whether the same sample was measured repeatedly
☐	☒	The statistical test(s) used AND whether they are one- or two-sided<br>*Only common tests should be described solely by name; describe more complex techniques in the Methods section.*
☐	☒	A description of all covariates tested
☐	☒	A description of any assumptions or corrections, such as tests of normality and adjustment for multiple comparisons
☐	☒	A full description of the statistical parameters including central tendency (e.g. means) or other basic estimates (e.g. regression coefficient) AND variation (e.g. standard deviation) or associated estimates of uncertainty (e.g. confidence intervals)
☐	☒	For null hypothesis testing, the test statistic (e.g. *F*, *t*, *r*) with confidence intervals, effect sizes, degrees of freedom and *P* value noted<br>*Give P values as exact values whenever suitable.*
☒	☐	For Bayesian analysis, information on the choice of priors and Markov chain Monte Carlo settings
☒	☐	For hierarchical and complex designs, identification of the appropriate level for tests and full reporting of outcomes
☒	☐	Estimates of effect sizes (e.g. Cohen's *d*, Pearson's *r*), indicating how they were calculated

Our web collection on statistics for biologists contains articles on many of the points above.

Software and code

Policy information about availability of computer code

Data collection	NA
Data analysis	R version 4.1.2

For manuscripts utilizing custom algorithms or software that are central to the research but not yet described in published literature, software must be made available to editors and reviewers. We strongly encourage code deposition in a community repository (e.g. GitHub). See the Nature Portfolio guidelines for submitting code & software for further information.

Data

Policy information about availability of data

All manuscripts must include a data availability statement. This statement should provide the following information, where applicable:

- Accession codes, unique identifiers, or web links for publicly available datasets
- A description of any restrictions on data availability
- For clinical datasets or third party data, please ensure that the statement adheres to our policy

We include the data and analysis R script in the supplementary files

March 2021

Human research participants

Policy information about studies involving human research participants and Sex and Gender in Research.

Reporting on sex and gender	*Use the terms sex (biological attribute) and gender (shaped by social and cultural circumstances) carefully in order to avoid confusing both terms. Indicate if findings apply to only one sex or gender; describe whether sex and gender were considered in study design whether sex and/or gender was determined based on self-reporting or assigned and methods used. Provide in the source data disaggregated sex and gender data where this information has been collected, and consent has been obtained for sharing of individual-level data; provide overall numbers in this Reporting Summary. Please state if this information has not been collected. Report sex- and gender-based analyses where performed, justify reasons for lack of sex- and gender-based analysis.*
Population characteristics	*Describe the covariate-relevant population characteristics of the human research participants (e.g. age, genotypic information, past and current diagnosis and treatment categories). If you filled out the behavioural & social sciences study design questions and have nothing to add here, write "See above."*
Recruitment	*Describe how participants were recruited. Outline any potential self-selection bias or other biases that may be present and how these are likely to impact results.*
Ethics oversight	*Identify the organization(s) that approved the study protocol.*

Note that full information on the approval of the study protocol must also be provided in the manuscript.

Field-specific reporting

Please select the one below that is the best fit for your research. If you are not sure, read the appropriate sections before making your selection.

☐ Life sciences ☐ Behavioural & social sciences ☒ Ecological, evolutionary & environmental sciences

For a reference copy of the document with all sections, see nature.com/documents/nr-reporting-summary-flat.pdf

Ecological, evolutionary & environmental sciences study design

All studies must disclose on these points even when the disclosure is negative.

Study description	The study measured the flight initiation distance of Eurasian curlew under a range of light conditions (both nocturnal and diurnal). FID was compared to light levels at the point of measurement. Covariates included group size, spatial location, tidal and weather variables.
Research sample	The study focused on Eurasian curlew overwintering on the Penryn mudflats. n = 86 nocturnal FID measurements and n = 27 diurnal FID measurements. The population of curlew were unmarked, and in any case it would have been impossible to identify individuals at night from a distance as the experimental design required working complete darkness with a thermal IR scope. As such there will be an unknown level of repeat sampling from the population (estimated at 50-70 individuals).
Sampling strategy	Curlew were identified and only selected when > 75m away. Curlew were sampled opportunistically, however the FIDs measured within a given night were all from different locations, and therefore unlikely to repeat-measure the same individual.
Data collection	Data were collected by JJ using a standard FID protocol.
Timing and spatial scale	Data were collected from October-December 2021. Data could only be collected when the tide was below 2.5m (based on Falmouth estimates). Specific light levels were targeted, e.g. new and full moon, cloudy and clear, ensuring a full range of conditions. However this was necessarily opportunistic. Data were not collected in high wind or rain.
Data exclusions	No data were excluded.
Reproducibility	No attempt at reproduction was made. We note that analyzing a subset of the data with a limited spatial scale and less direct artificial light at night showed the same trends as the full dataset.
Randomization	The study was field based, with numerous uncontrolled variables. These were included as covariates in statistical models (location, group size, tide range, tide height, temperature, and date), with light levels as the main explanatory variable. Sampling was random.
Blinding	Blinding was not possible (the experimenter could not have been made blind to the light levels at each FID event). However, light levels (which have little room for experimenter bias) were only measured after the FID measurement (which could feasibly be more prone to experimenter bias). As such, for more measurements the experimenter would have been unaware of the exact absolute light levels.

Did the study involve field work? ☒ Yes ☐ No

Field work, collection and transport

Field conditions	These were measured and were included as covariates, available in the dataset
Location	Penryn mudflats centred on 50°10’05.4”N, 5°05’29.7”W
Access & import/export	no permits were required (public access), no birds were handled
Disturbance	3 Rs (reduce, replace, refine) were considered in our full ethics application. no more than 5 FIDs were measured per night (to minimize disturbance). We also chose a target sample size that would be able to detect a moderate statistical effect, while reducing the amount of disturbance to a locally endangered species.

Reporting for specific materials, systems and methods

We require information from authors about some types of materials, experimental systems and methods used in many studies. Here, indicate whether each material, system or method listed is relevant to your study. If you are not sure if a list item applies to your research, read the appropriate section before selecting a response.

Materials & experimental systems

n/a	Involved in the study
☒	☐ Antibodies
☒	☐ Eukaryotic cell lines
☒	☐ Palaeontology and archaeology
☐	☒ Animals and other organisms
☒	☐ Clinical data
☒	☐ Dual use research of concern

Methods

n/a	Involved in the study
☒	☐ ChIP-seq
☒	☐ Flow cytometry
☒	☐ MRI-based neuroimaging

Animals and other research organisms

Policy information about studies involving animals; ARRIVE guidelines recommended for reporting animal research, and Sex and Gender in Research

Laboratory animals	NA
Wild animals	A wild population of Eurasian curlew Numenius arquata were studied. The individuals were unmarked, and the experiment did not involve trapping or handling any animals.
Reporting on sex	Unknown.
Field-collected samples	NA
Ethics oversight	Ethical approval was secured from Exeter University (CLES Cornwall Ethics Committee, ID 493710)

Note that full information on the approval of the study protocol must also be provided in the manuscript.
